# Supplementary material for: Simulation Studies Provide Evidence of Aerosol Transmission of SARS-CoV-2 in a Multi-Story Building via Air Supply, Exhaust and Sanitary Pipelines
Source: Int J Environ Res Public Health. 2022 Jan 29;19(3):1532. doi: 10.3390/ijerph19031532 (PMC8835679; doi:10.3390/ijerph19031532)
Supplement: Supplementary file 1 [file ijerph-19-01532-s001.zip › ijerph-1522993-supplementary.pdf]

Table S1. The method, time, sample location in different rooms for the aerosol generation, monitor and acquisition in the scenario of toilet flushing and respiration simulation.

**Aerosol Generation**							**Monitor and Acquisition**			
**Scenario 1**					Scenario 2				Scenario 1	Scenario 2
**Room\ Time**	**0h**	**0.5h**	**1h**	1.5h	Room \ Time	0-4h	Method	Sample Location	Acquisition Time	Acquisition Time
552	Pouring simulants and toilet flushing	Toilet flushing	Pouring simulants and toilet flushing	Toilet flushing	510	Simulating respiration to generate aerosols	Concentration monitor: particle size spectrometer, PM_{10} measuring instrument	Bathroom	Every 10 minutes	Every 30 minutes
								Bedroom	/	Every 10 minutes
								Corridor	/	Every 1 hour
							Smear sampling	Floor drain, toilet lid and seat ring	Every 30 minutes	/
652	Flushing simultaneously	Flushing simultaneously	Flushing simultaneously	Flushing simultaneously	511	Simulating respiration to generate aerosols		Exhaust fan	Every 30 minutes	/
								Air supply outlets of fresh air system	Every 30 minutes	Every 1 hour
								Air supply outlet of the air conditioning		Every 1 hour
								Surface of the table, door, bed	/	Every 1 hour
752	Flushing simultaneously	Flushing simultaneously	Flushing simultaneously	Flushing simultaneously	552	Simulating respiration to generate aerosols		Exhaust outlet of the roof	After the experiment	After the experiment
								Sewage pipe riser vent on the roof	After the experiment	After the experiment
							Air suspension sample collection : filter membrane sampler, bioaerosol sampler	Bathroom	0-1.5h	/
								Bedroom	/	0-2h,2-4h

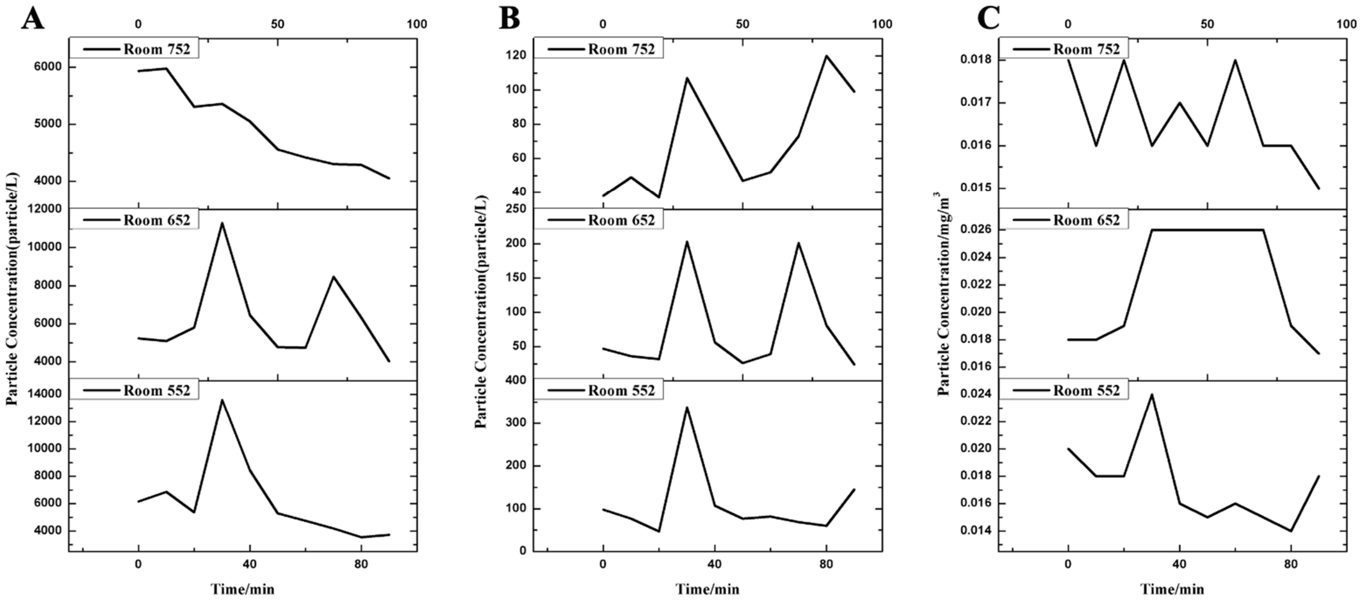

Figure S1. The changes of particle concentration over time in 3 different rooms (Room 552, 652, 752) in the scenario of toilet flushing. A.0.5μm; B.5μm; C.PM10.

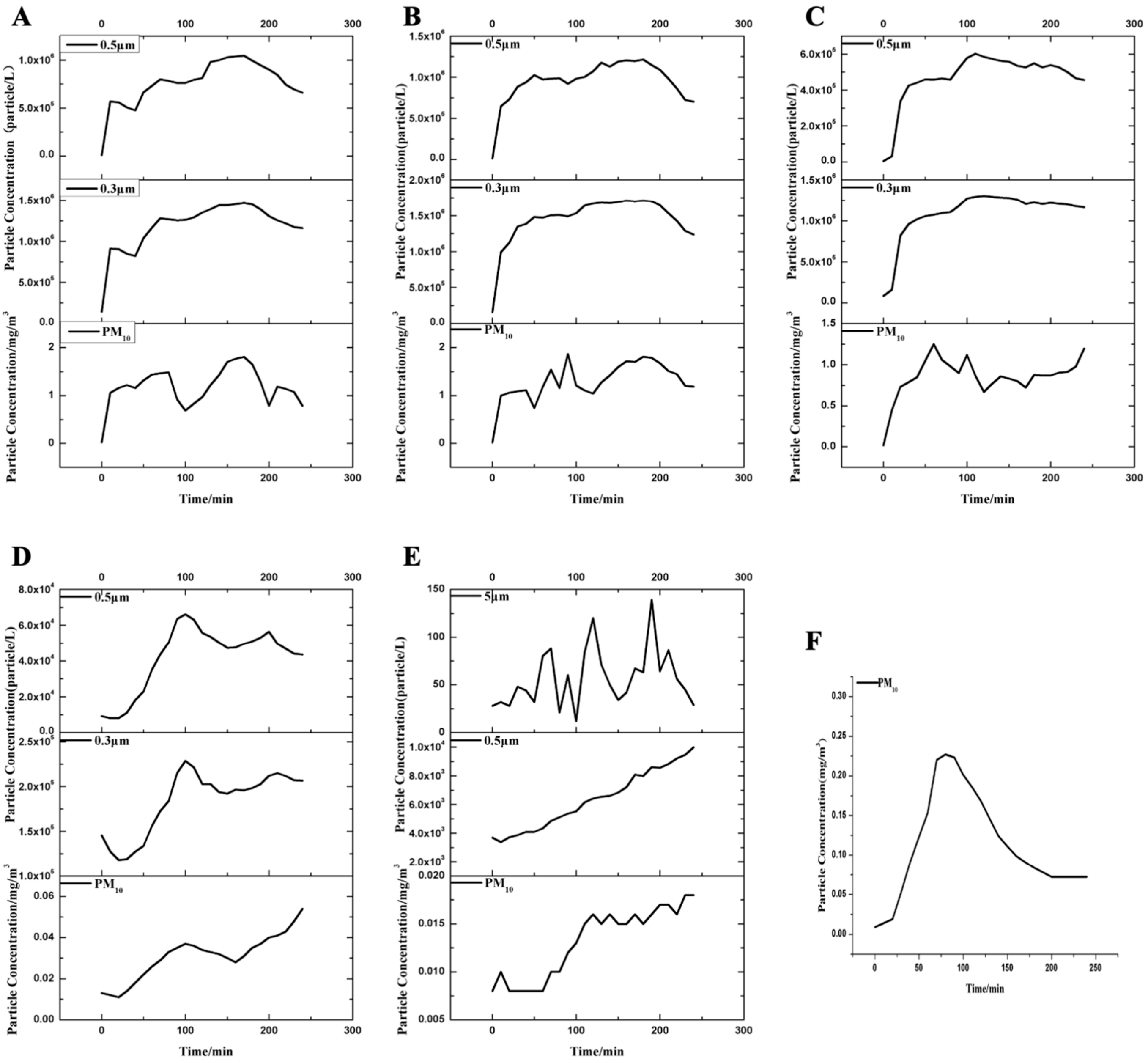

Figure S2. The changes of particle concentration over time at 0.3μm, 0.5μm, 5μm, PM_{10} in different rooms in the scenario of respiration simulation. A.Room 510; B.Room 511; C.Room 552. D.Room 652; E.Room 705; F.Room 553.

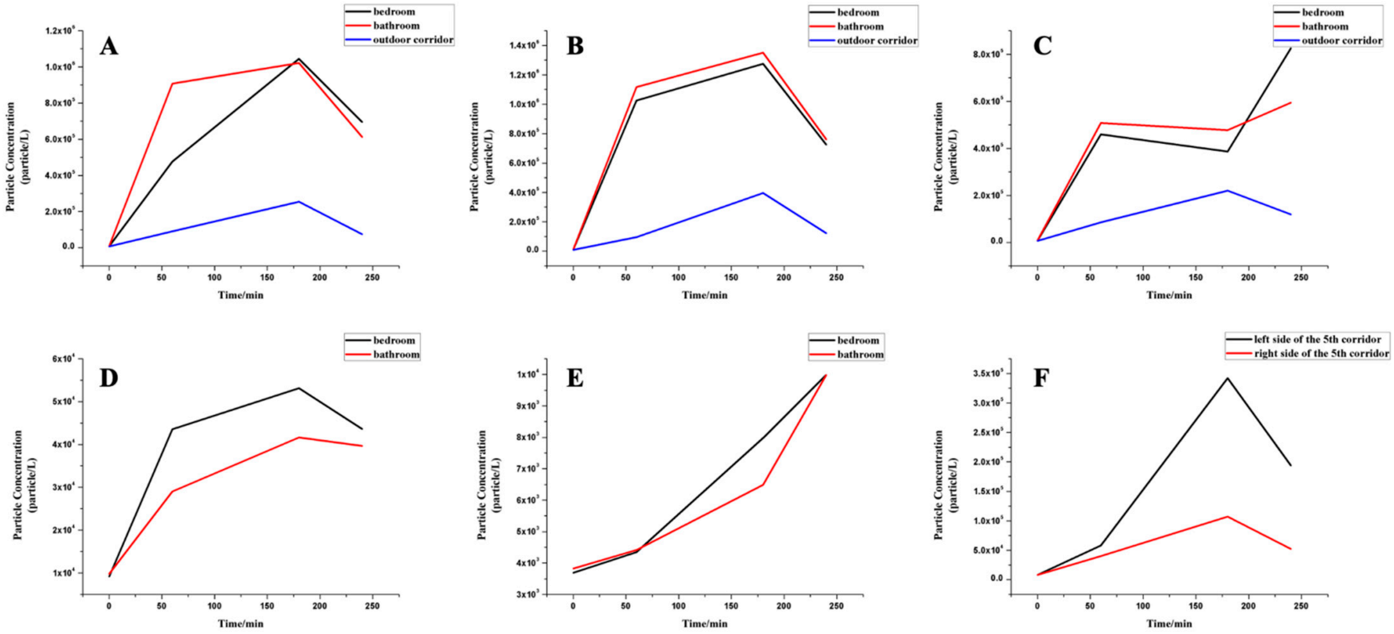

Figure S3. The changes of particle concentration at 0, 60, 180, 240 min at 0.5μm in the bedroom, bathroom, outdoor corridor of different rooms in the scenario of respiration simulation. A.Room 510; B.Room 511; C.Room 552. D.Room 652; E.Room 705; F.The 5th corridor.

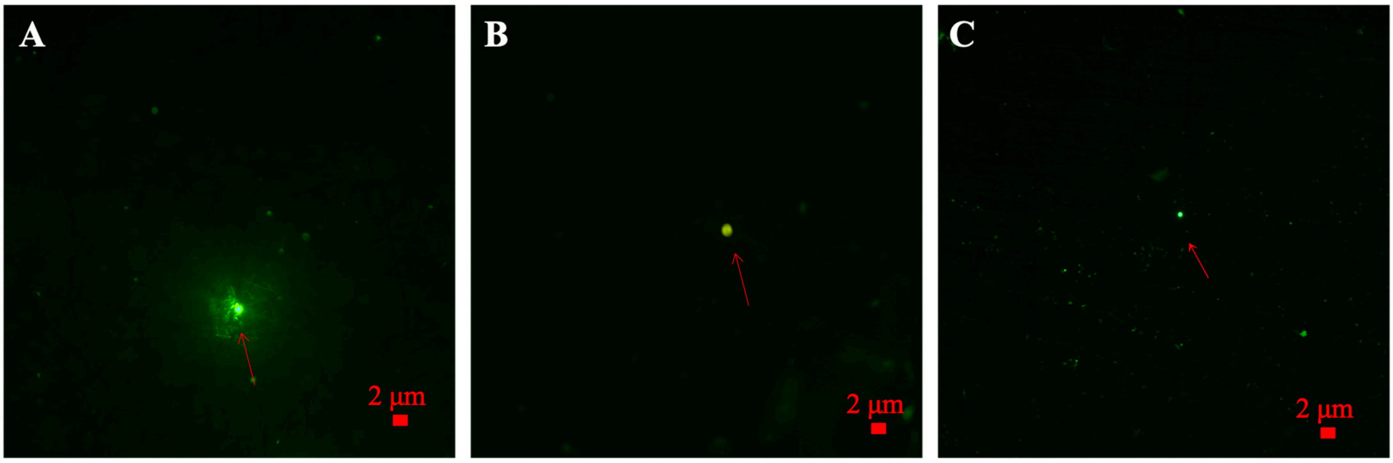

Figure S4. Representative photos of fluorescent microspheres tracked by different sampling methods in different sites. After toilet flushing and respiration simulation, fluorescent microspheres (yellow and green) were detected in (A) the aerosol filter membrane sample using PM_{10} samplers (100 L/min) under fluorescence microscopy; (B) the aerosol liquid. sample using biological aerosol samplers (100 L/min) under fluorescence microscopy; and (C) an air sample using natural sedimentation on floor drain, toilet lid and seat ring; table, door, bed; exhaust fan of bathroom; air supply outlet etc., under fluorescence microscopy.
