# Supplementary material for: Simulation Studies Provide Evidence of Aerosol Transmission of SARS-CoV-2 in a Multi-Story Building via Air Supply, Exhaust and Sanitary Pipelines
Source: Int J Environ Res Public Health. 2022 Jan 29;19(3):1532. doi: 10.3390/ijerph19031532 (PMC8835679; doi:10.3390/ijerph19031532)
Supplement: Supplementary file 1 [file ijerph-19-01532-s001.zip › raw data.pdf]

Figure 1. A data The changes of particle concentration over time at 0.5μm (scenarios 1), PM_{10} (scenarios 2) in different rooms . A.scenarios 1; B.scenarios 2.

	Particle Concentration/5μm/particle/L		
**Time/min**	**Room 552**	**Room 652**	**Room 752**
0	98	47	38
10	77	36	49
20	47	32	37
30	338	203	107
40	107	56	77
50	77	26	47
60	82	39	52
70	69	201	73
80	60	81	120
90	145	24	99

Figure 1. B data The changes of particle concentration over time at 0.5μm (scenarios 1), PM_{10} (scenarios 2) in different rooms . A.scenarios 1; B.scenarios 2.

	Particle Concentration/PM_{10}/mg/m^3					
**Time/min**	**Room 510**	**Room 511**	**Room 552**	**Room 553**	**Room 652**	**Room 705**
0	0.02	0.016	0.016	0.009	0.013	0.008
10	1.059	1.001	0.449	0.014	0.012	0.01
20	1.162	1.061	0.732	0.019	0.011	0.008
30	1.219	1.089	0.789	0.054	0.014	0.008
40	1.161	1.112	0.849	0.091	0.018	0.008
50	1.319	0.744	1.05	0.123	0.022	0.008
60	1.44	1.183	1.25	0.154	0.026	0.008
70	1.465	1.54	1.06	0.22	0.029	0.01
80	1.484	1.16	0.981	0.227	0.033	0.01
90	0.925	1.868	0.901	0.223	0.035	0.012
100	0.69	1.21	1.12	0.201	0.037	0.013
110	0.834	1.108	0.864	0.186	0.036	0.015
120	0.968	1.046	0.67	0.168	0.034	0.016

130	1.221	1.275	0.771	0.146	0.033	0.015
140	1.425	1.423	0.858	0.124	0.032	0.016
150	1.706	1.598	0.832	0.111	0.03	0.015
160	1.768	1.717	0.803	0.099	0.028	0.015
170	1.805	1.704	0.725	0.091	0.031	0.016
180	1.654	1.811	0.874	0.084	0.035	0.015
190	1.276	1.79	0.872	0.078	0.037	0.016
200	0.789	1.676	0.87	0.072	0.04	0.017
210	1.188	1.511	0.905	0.072	0.041	0.017
220	1.14	1.448	0.914	0.072	0.043	0.016
230	1.077	1.209	0.98	0.072	0.048	0.018
240	0.79	1.187	1.2	0.072	0.054	0.018

Figure S1. data The variation of particle concentration (0.5μm, 5μm, PM_{10}) over time in different rooms in the scenario of toilet flushing.

Time/min	Particle Concentration								
	0.5μm/particle/L			5μm/particle/L			PM_{10}/mg/m^3		
	Room 552	Room 652	Room 752	Room 552	Room 652	Room 752	Room 552	Room 652	Room 752
0	6166	5235	5934	98	47	38	0.02	0.018	0.018
10	6857	5094	5977	77	36	49	0.018	0.018	0.016
20	5371	5808	5306	47	32	37	0.018	0.019	0.018
30	13612	11288	5357	338	203	107	0.024	0.026	0.016
40	8437	6443	5053	107	56	77	0.016	0.026	0.017
50	5286	4778	4560	77	26	47	0.015	0.026	0.016
60	4758	4748	4421	82	39	52	0.016	0.026	0.018
70	4182	8465	4305	69	201	73	0.015	0.026	0.016
80	3553	6314	4288	60	81	120	0.014	0.019	0.016
90	3722	4029	4054	145	24	99	0.018	0.017	0.015

Figure S2. data. The changes of particle concentration over time at 0.3μm, 0.5μm, 5μm, PM10 in different rooms in the scenario of respiration simulation.

Time/ min	Particle Concentration															
	Room510			Room511			Room552			Room652			Room705			Room 553
	PM_{10}/ mg/m^3	0.3μm/particle/L	0.5μm/particle/L	PM_{10}/ mg/m^3	0.3μm/ parti-cle/L	0.5μm/ parti-cle/L	PM_{10}/ mg/m^3	0.3μm/ parti-cle/L	0.5μm/ parti-cle/L	PM_{10}/ mg/m^3	0.3μm/ parti-cle/L	0.5μm/ parti-cle/L	PM_{10}/ mg/m^3	0.5μm/ parti-cle/L	5μm/particle/L	PM_{10}/ mg/m^3
0	0.02	141635	8720	0.016	158302	11992	0.016	86589	4673	0.013	145626	9175	0.008	3694	28	0.009
10	1.059	910572	569876	1.001	990742	645957	0.449	161423	31983	0.012	127724	8163	0.01	3391	32	0.014
20	1.162	904956	559223	1.061	1122566	731502	0.732	816957	337421	0.011	117946	8153	0.008	3717	28	0.019
30	1.219	849020	506719	1.089	1348875	885438	0.789	957718	424519	0.014	119096	11055	0.008	3857	48	0.054
40	1.161	820798	476423	1.112	1385195	937887	0.849	1018764	441343	0.018	127350	18125	0.008	4100	44	0.091
50	1.319	1038594	664463	0.744	1480621	1025343	1.05	1056363	459163	0.022	133875	23026	0.008	4103	32	0.123
60	1.44	1165221	730787	1.183	1470474	975053	1.25	1073958	458395	0.026	156283	35166	0.008	4345	80	0.154
70	1.465	1278016	800447	1.54	1504833	980556	1.06	1092979	463866	0.029	172632	43530	0.01	4859	88	0.22

80	1.484	1268194	783004	1.16	1509983	987687	0.981	1105066	458781	0.033	183933	50338	0.01	5113	21	0.227
90	0.925	1255695	761298	1.868	1491455	920870	0.901	1180743	521484	0.035	214961	63471	0.012	5363	60	0.223
100	0.69	1259902	763526	1.21	1532675	983052	1.12	1273956	578447	0.037	228581	66193	0.013	5523	12	0.201
110	0.834	1289204	793556	1.108	1639982	1004560	0.864	1295807	602058	0.036	221476	63140	0.015	6157	84	0.186
120	0.968	1349683	813349	1.046	1671468	1072146	0.67	1302512	584607	0.034	202900	55898	0.016	6417	120	0.168
130	1.221	1391184	980017	1.275	1683372	1178977	0.771	1293849	573556	0.033	202819	53589	0.015	6548	71	0.146
140	1.425	1439021	998157	1.423	1673998	1129328	0.858	1284039	562387	0.032	193911	50429	0.016	6606	50	0.124
150	1.706	1440092	1029984	1.598	1690983	1190324	0.832	1279302	557645	0.03	192148	47261	0.015	6834	34	0.111
160	1.768	1452386	1037897	1.717	1709385	1203385	0.803	1259830	535238	0.028	196537	47600	0.015	7214	42	0.099
170	1.805	1466183	1044904	1.704	1700832	1196609	0.725	1205165	526680	0.031	195750	49587	0.016	8070	67	0.091
180	1.654	1449604	993750	1.811	1710654	1214796	0.874	1223943	548967	0.035	198368	51058	0.015	7982	63	0.084
190	1.276	1387750	946785	1.79	1702231	1147732	0.872	1205738	526755	0.037	202693	53127	0.016	8606	139	0.078
200	0.789	1307192	902418	1.	164309	108907	0.87	121948	538986	0.04	211955	56436	0.017	8559	64	0.072

				676	9	7		3								
210	1.188	1255023	845773	1.511	1532265	982320	0.905	1208964	526722	0.041	215093	49642	0.017	8822	86	0.072
220	1.14	1215167	744562	1.448	1426932	863354	0.914	1199662	498476	0.043	211843	46755	0.016	9200	56	0.072
230	1.077	1174224	695664	1.209	1287010	725231	0.98	1177920	465733	0.048	207093	44016	0.018	9462	45	0.072
240	0.79	1160098	658834	1.187	1234991	701922	1.2	1164324	455223	0.054	206554	43590	0.018	9985	29	0.072

Figure S3. data. The changes of particle concentration at 0, 60, 180, 240 min at 0.5μm in the bedroom, bathroom, outdoor corridor of different rooms in the scenario of respiration simulation.

Time/ min	Particle Concentration/0.5μm/particle/L														
	**Room510**			**Room511**			**Room552**			**Room652**		**Room705**		**5th corridor**	
	**bed-room**	**bath-room**	**outdoor corridor**	**bed-room**	**bath-room**	**outdoor corridor**	**bed-room**	**bath-room**	**outdoor corridor**	**bed-room**	**bath-room**	**bed-room**	**bath-room**	**left side**	**right side**
0	8720	9035	8291	11992	12178	8744	7618	8371	7033	9175	9822	3694	3822	8035	8311
60	476423	907676	91442	1025343	1116854	96565	459163	507986	84862	43530	29044	4345	4415	58080	40101
180	1044904	1021767	255410	1274796	1351436	395777	386680	477349	219625	53127	41610	7982	6489	342228	106935
240	695664	612090	75738	725231	761619	123078	825733	593950	118667	43590	39668	9985	9985	193772	52249
